# Linkage Mapping and Comparative Genomics Using Next-Generation RAD Sequencing of a Non-Model Organism

**DOI:** 10.1371/journal.pone.0019315

**Published:** 2011-04-26

**Authors:** Simon W. Baxter, John W. Davey, J. Spencer Johnston, Anthony M. Shelton, David G. Heckel, Chris D. Jiggins, Mark L. Blaxter

**Affiliations:** 1 Department of Zoology, University of Cambridge, Cambridge, United Kingdom; 2 Institute of Evolutionary Biology, University of Edinburgh, Edinburgh, United Kingdom; 3 Department of Entomology, Texas A&M University, College Station, Texas, United States of America; 4 Department of Entomology, Cornell University, New York State Agricultural Experiment Station, Geneva, New York, United States of America; 5 Department of Entomology, Max Planck Institute for Chemical Ecology, Jena, Germany; 6 The GenePool Genomics Facility, Institute of Evolutionary Biology, University of Edinburgh, Edinburgh, United Kingdom; University of Umeå, Sweden

## Abstract

Restriction-site associated DNA (RAD) sequencing is a powerful new method for targeted sequencing across the genomes of many individuals. This approach has broad potential for genetic analysis of non-model organisms including genotype-phenotype association mapping, phylogeography, population genetics and scaffolding genome assemblies through linkage mapping. We constructed a RAD library using genomic DNA from a *Plutella xylostella* (diamondback moth) backcross that segregated for resistance to the insecticide spinosad. Sequencing of 24 individuals was performed on a single Illumina GAIIx lane (51 base paired-end reads). Taking advantage of the lack of crossing over in homologous chromosomes in female Lepidoptera, 3,177 maternally inherited RAD alleles were assigned to the 31 chromosomes, enabling identification of the spinosad resistance and W/Z sex chromosomes. Paired-end reads for each RAD allele were assembled into contigs and compared to the genome of *Bombyx mori* (n = 28) using BLAST, revealing 28 homologous matches plus 3 expected fusion/breakage events which account for the difference in chromosome number. A genome-wide linkage map (1292 cM) was inferred with 2,878 segregating RAD alleles inherited from the backcross father, producing chromosome and location specific sequenced RAD markers. Here we have used RAD sequencing to construct a genetic linkage map *de novo* for an organism that has no previous genome data. Comparative analysis of *P. xyloxtella* linkage groups with *B. mori* chromosomes shows for the first time, genetic synteny appears common beyond the Macrolepidoptera. RAD sequencing is a powerful system capable of rapidly generating chromosome specific data for non-model organisms.

## Introduction

Discovering genes that control morphological, behavioural and physiological phenotypes is critical for understanding adaptive evolution, for plant and animal breeding and for tracking the evolutionary responses of natural populations, such as insecticide resistance in crop pests. Commonly, traits controlled by single, major Mendelian genes are isolated using genetic linkage maps created from crossing experiments. A genome-wide analysis is followed by finer scale mapping with a larger number of recombinant individuals to narrow the region of interest, and finally targeted sequencing of genome libraries. Linkage maps have been constructed for scores of organisms, generally to identify a genome region controlling a trait of interest, such as skeletal armour morphology in stickleback fish [1], [2], wing patterns in butterflies [3], [4] and morphological and physiological traits in sunflowers [5]. Amplified fragment length polymorphism (AFLP), RAPDs, microsatellites and single copy gene markers used to construct such maps are all problematic, either being expensive and difficult to develop, or anonymous and difficult to translate into useful sequence-based markers. Next-generation sequencing can now greatly facilitate the process of genetic mapping, allowing rapid generation of dense genome linkage maps consisting of thousands of sequenced markers, such that useful sequences linked to a gene of interest can be identified in a single experiment.

The restriction site associated DNA (RAD) sequencing method [6], [7], [8], [9] facilitates genetic variant discovery by sequencing only the DNA flanking specific restriction enzyme sites, allowing orthologous sequences to be targeted in multiple individuals. The method relies on cutting DNA with a chosen restriction enzyme, ligating an adapter containing a molecular identifying sequence (MID) unique to each sample, and sequencing the DNA associated with each restriction site using the massively parallel Illumina sequencing technology [10]. The method has proven highly successful in re-identifying genomic regions controlling known phenotypes [6], [11] and comparing adaptive evolution between populations of organisms with [9] and without [8] reference genomes.

*Plutella xylostella*, the diamondback moth, is a worldwide pest of cruciferous crops including cabbage and broccoli [12]. Resistances to insecticides have been independently reported from multiple, isolated populations [13], [14]. Recently, we identified a candidate resistance allele that confers high level resistance to the insecticide spinosad, using AFLP genetic mapping and single copy gene anchors [15]. Here, we use RAD genotyping to (i) re-identify the spinosad resistance chromosome, (ii) assign thousands of RAD alleles to specific *P. xylostella* chromosomes, (iii) compare chromosomal synteny with the reference lepidopteran genome from the silkworm *Bombyx mori,* (iv) identify RAD loci likely to encode transcribed genes and (v) create a genome-wide linkage map with sequenced RAD loci. Instead of mapping the RAD tags directly to a reference genome, we constructed contigs using paired-end sequences adjacent to the RAD tags and used these for gene identification and interspecific comparisons. The methods described have great potential for creating genomic scaffolds to assist in genome assembly and for identifying thousands of sequence variants to aid in detection of major as well as minor quantitative traits.

## Results

### Processing RAD sequences

Resistance to the insecticide spinosad is recessive and controlled by a single Mendelian locus in the *P. xylostella* strain Pearl-Sel [14], [15]. The resistance locus has been mapped to a chromosomal region containing a truncating point mutation in a putative receptor; nicotinic acetylcholine receptor alpha 6 [15]. We constructed a backcross pedigree, crossing a spinosad-resistant Pearl-Sel male with a spinosad-sensitive (Pearl-Sel x Geneva88) F_1_ female. Genotyping of known markers confirmed all 12 of 12 spinosad survivors and 7 of 10 untreated controls were homozygous for the resistance mutation. A single RAD library was constructed from DNA of the two backcross parents and 22 backcross progeny (10 untreated controls and 12 that had survived the spinosad challenge). We estimated that there should be ∼6500 Sbf1 RAD loci in the *P. xylostella* genome, and thus generated raw Illumina reads to a depth of 65x per locus per individual based on this estimate. This was achieved in a single Illumina GAIIx lane, producing 10,217,074 51-base paired-end reads. Each forward sequence contained a 5 base MID that allowed reads to be assigned to a specific individual, followed by 6 bases of the SbfI restriction enzyme footprint (TGCAGG), leaving 40 locus-specific bases per read.

The sequence reads were split into groups by MID, resulting in one group for each of the 24 individual moths. The average number of sequence reads per individual was 425,000, although there was high variability amongst the 24 individuals (16K-842K) (Table 1). Two of the progeny (untreated control 5 and spinosad survivor 12) had significantly fewer RAD reads than the average and were removed from the analysis, raising the average number of reads per individual to 461,500. For each individual, the 40-base reads were clustered into candidate RAD loci containing one or more candidate RAD alleles. These RAD loci were then merged across all individuals, with RAD alleles appearing in only one individual removed from the analysis. 8,342 candidate RAD loci were identified, containing 13,768 candidate RAD alleles. Two RAD haplotypes were considered allelic if they had 3 or fewer base differences within the 40 bases of forward sequence. This candidate set is liberally defined to avoid losing any real RAD alleles, with a subset of RAD alleles selected later based on linkage, as described below.

**Table 1:** Number of Illumina reads sequenced per individual.

Number of Illumina reads sequenced per individual				Coverage per RAD allele	
Individual	Sex	MID	Sequences	Mean	SD
Father	Male	CGATA	297,795	27.9	17.7
Mother	Female	CGGCG	495,190	41.9	17.5
Control 1	Female	CTAGG	474,048	43.1	17.8
Control 2	Male	CTGAA	381,912	34.5	16.2
Control 3	Female	GAAGC	467,094	42.5	17.2
Control 4	Male	GAGAT	496,749	43.5	20.1
Control 5	Male	GCATT	16,959	-	-
Control 6	Male	GGAAG	510,666	45.4	18.5
Control 7	Male	GTACA	842,036	69.4	35.1
Control 8	Female	TAATG	414,966	38.4	16.8
Control 9	Female	TAGCA	462,595	42.4	18.2
Control 10	Female	TCAGA	325,581	30.7	13.5
Bioassay 1	Male	TCGAG	510,086	46.3	17.7
Bioassay 2	Male	TGACC	609,811	48.8	24.6
Bioassay 3	Male	TGGTT	374,328	34.7	15.1
Bioassay 4	Male	TTAAT	168,157	15.9	8.1
Bioassay 5	Male	AACCC	375,236	34.5	14.1
Bioassay 6	Female	ACTGC	373,667	33.8	14.6
Bioassay 7	Female	AAGGG	513,757	44.2	20.2
Bioassay 8	Female	ACGTA	463,518	43.2	17.6
Bioassay 9	Male	AGAGT	638,261	58.4	24.6
Bioassay 10	Male	ATGCT	482,003	43.7	20.6
Bioassay 11	Male	CAGTC	476,704	40.7	22.1
Bioassay 12	Female	CCAAC	45,955	-	-
All (24)	14M, 10F	-	Mean 425,711	-	-
Analysed (22)	13M, 9F	-	Mean 461,553	41.1	18.5

24 individuals were multiplexed on one lane of Illumina GAIIx sequencing, yielding 10,217,074 reads (mean 425,711). The above table lists the numbers of reads featuring the molecular identifiers (MIDs) assigned to each individual. Individuals ‘Control 5’ and ‘Bioassay 12’ were excluded from further analyses due to the low read counts for these individuals, resulting in an average read number of 461,533. Mean+/−SD read counts for high quality RAD alleles (3177 maternal-derived and 2878 paternal-derived, 6055 in total; see Table 3) are given.

**Figure 1:**
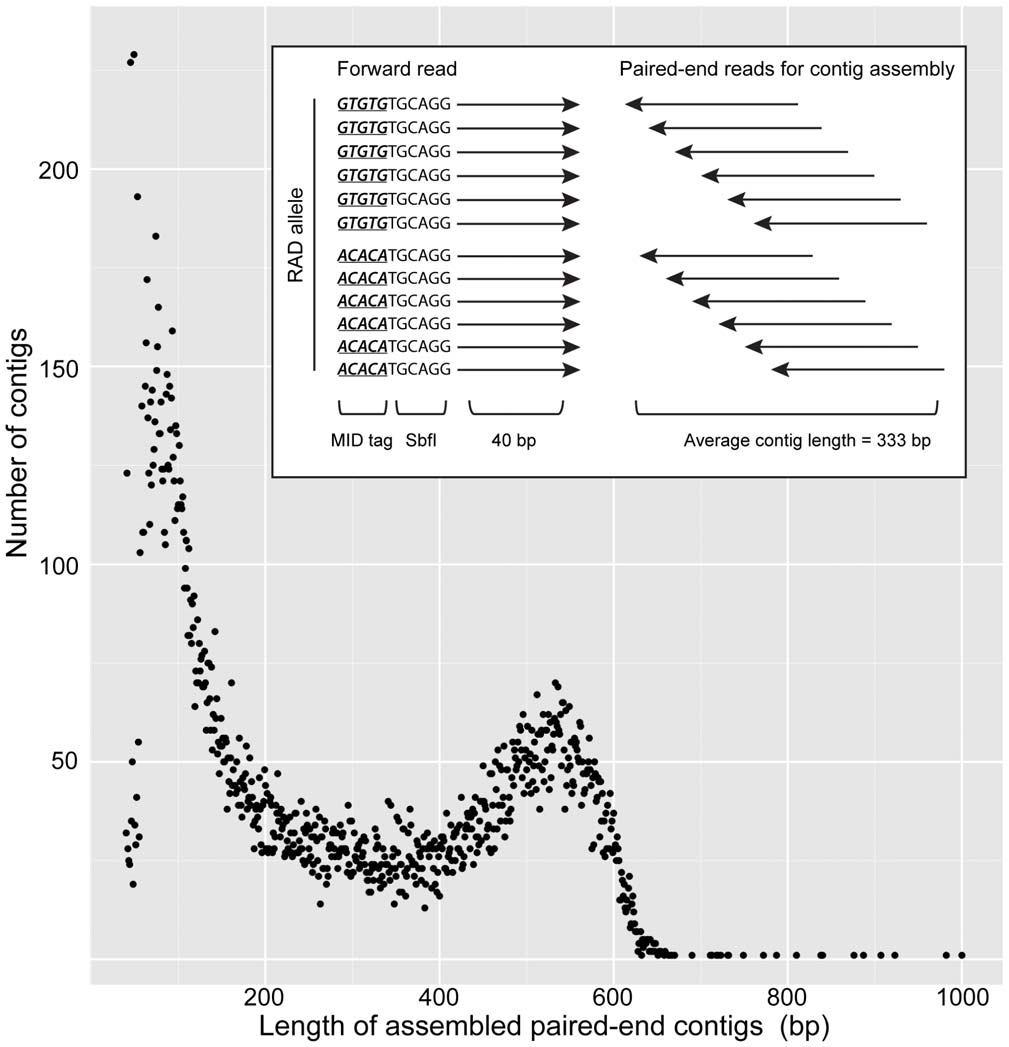
Assembling RAD alleles and creating RAD contigs from RAD paired-end reads. **Inset**. Each forward sequence read contains a 5 base molecular identifier (MID), followed by 6 bp of SbfI restriction enzyme footprint. After identifying candidate RAD alleles, RAD contigs were assembled from the paired-end reads. **Scatter plot**. The size distribution of RAD assembled paired-end contigs. The X-axis is contig length in base pairs and Y-axis the number of RAD contigs with each length. The mean read length is 333 bases. One RAD allele can have multiple RAD paired-end contigs, as significant variation in the paired-end sequence can result in assembly of more than one contig, or low coverage may prevent complete assembly. Read lengths of 57 bases (containing 542 contigs) and 61 bases (containing 596 contigs) plus 200 contigs containing stretches of Ns were deemed to be assembly artefacts and omitted.

The forward sequence for each RAD allele begins at a defined position; the SbfI cut site. Due to size shearing of the library, the paired-end reverse sequence read for individual RAD alleles was located a variable distance from the cut site, and could be grouped and clustered into RAD contigs using VelvetOptimiser. The length distribution of RAD contigs produced was bimodal, with average length  = 333 bp, median length  = 211 bp (Figure 1). RAD contigs do not overlap the 40-base RAD allele but are expected to be spaced within 300–700 bp of them in the genome (as determined by the size of the sheared genomic DNA). Thus, RAD contigs could be used in BLAST searches for homologous genes in other species, which would not have been possible with the 40-base RAD alleles themselves.

### Mapping maternally derived RAD alleles to linkage groups

Crossing over between homologous chromosomes does not occur in female Lepidoptera. Hence all genes and markers on the same maternally inherited chromosome are completely linked [16], [17]. *P. xylostella* (2n = 62) backcross progeny therefore inherit one unrecombined chromosome of each maternal pair. Crossing over does occur during spermatogenesis in males, enabling the 31 paternally inherited chromosomes to be used for linkage mapping and determining genetic distances between markers [4].

To identify RAD alleles specific to each chromosome, candidate RAD alleles present in the mother and absent from the father were analyzed. A subset of these RAD alleles segregated (present or absent) across the 20 backcross progeny, indicating that the F_1_ mother was heterozygous. The presence/absence of candidate RAD alleles in a set of progeny individuals can be represented as a binary number string of 1 (allele present) or 0 (allele absent) per individual. For 20 progeny, more than 1 million combinations are mathematically achievable (2^20^). However, we expect to find 62 patterns representing segregation of the 31 maternal chromosome pairs. From 4,826 candidate maternally derived RAD alleles, 3,177 were grouped into exactly 31 pairs of different binary patterns, each representing a distinct chromosome containing 49–190 sequenced markers (mean = 102.5 per chromosome, Table 2). A further 223 candidate RAD alleles were present in the mother and all offspring but not the father, and so are likely to be homozygous in the mother. The remaining 1,426 candidate RAD alleles grouped into 1,055 binary patterns and are likely to be a result of a combination of sampling and technical error. Most were almost identical to one of the 62 patterns of interest but with genotypes from one or more individuals missing (i.e. false-negative allele nulls). Others likely arise from the presence of repeated sequence in the genome, and errors in sequencing. Each of these likely error patterns occurred in less than 20 candidate RAD alleles and 914 patterns were associated with a single candidate RAD allele. These errors are not unexpected given the varying coverage across the individual moths.

**Table 2:** Segregation Patterns and RAD Alleles for *P. xylostella* linkage groups.

LG	Segregation Pattern 1	Alleles	Segregation Pattern 2	Alleles	Total
1	010111000 11111000111	65	101000111 00000111000	39	104
2	001010110 11101001010	25	110101001 00010110101	24	49
3	101100000 00111110011	64	010011111 11000001100	50	114
4	000001010 11111101101	104	111110101 00000010010	56	160
5	100000110 00000000000	108	011111001 11111111111	82	190
6	010011101 11000110001	46	101100010 00111001110	43	89
7	111110000 10100101000	41	000001111 01011010111	34	75
8	001101010 01001010000	76	110010101 10110101111	70	146
9	100011010 11110011101	41	011100101 00001100010	30	71
10	000111001 11100011000	81	111000110 00011100111	57	138
11	110001111 11000111100	40	001110000 00111000011	29	69
12	011100001 10100011010	53	100011110 01011100101	45	98
13	110100001 00011111000	73	001011110 11100000111	39	112
14	101011101 10011101000	32	010100010 01100010111	28	60
15	110111110 01100011101	77	001000001 10011100010	52	129
16	011010010 10110000001	80	100101101 01001111110	70	150
17	101100011 00111001011	73	010011100 11000110100	69	142
18	111100101 11101011000	38	000011010 00010100111	38	76
19	011001111 11010010111	46	100110000 00101101000	40	86
20	011101011 11001100010	57	100010100 00110011101	44	101
21	011000100 11001100001	78	100111011 00110011110	26	104
22	000100010 00100111001	54	111011101 11011000110	44	98
23	101011100 10010111001	64	010100011 01101000110	44	108
24	000100111 11101100101	41	111011000 00010011010	36	77
25	101110101 00001010101	77	010001010 11110101010	56	133
26	101111110 10100101101	51	010000001 01011010010	24	75
27	100111010 11011101000	70	011000101 00100010111	35	105
28	100011011 10100001000	47	011100100 01011110111	41	88
29	001111110 10001001010	52	110000001 01110110101	47	99
30	000111000 00111111100	46	111000111 11000000011	32	78
31	011010100 10111000010	28	100101011 01000111101	25	53
Total		1828		1349	3177

Maternally derived segregation patterns (for RAD alleles present in the mother but absent in the father) for the 62 chromosomes of *P. xylostella* fall into 31 pairs. Offspring segregation patterns are presented as binary strings, where 1 indicates presence of RAD allele and 0 indicates absence. The two sets of digits separated by a space are the Control and Bioassay individuals (Control 5 and Bioassay 12 are not included). For example, Segregation Pattern 1 for PxLG5 (the resistance chromosome) is present in Controls 1, 8 and 9 and absent in all other individuals, including all Bioassay survivors. 108 candidate RAD alleles had this segregation pattern, with 82 candidate RAD alleles having the inverse pattern. The segregation patterns for PxLG1 identify the sex chromosomes (sexes segregate FMFMMMFFFMMMMMFFFMMM; see Table 1). Segregation Pattern 2 is the W chromosome (present in females, absent in males) and Segregation Pattern 1 the Z chromosome.

### Chromosomal synteny in Lepidoptera

Lepidoptera show a high degree of genetic synteny [18], but local rearrangements appear common [19]. To enable a comparative analysis, the RAD contigs associated with the 3,177 maternally derived, chromosome-linked RAD alleles were compared using TBLASTX to the 28 sequenced chromosomes of *Bombyx mori* (432 Mb) using an expect value cut-off of <1E-10. RAD contigs with hits to more than one chromosome were removed from the analysis. From each *P. xylostella* linkage group, the number of significant hits to each *B. mori* chromosome were counted to identify the most likely chromosomal homologue. Due to differences in chromosome number, it was expected that multiple *P. xylostella* chromosomes would collapse into a single *B. mori* chromosome. The 31 linkage groups collapsed into the 28 chromosomes of *B. mori*, and have been numbered according to *B. mori* chromosome nomenclature with three exceptions: *B. mori* chromosome (BmChr) 11 represents a fusion of *P. xylostella* linkage group (PxLG) 11 and 29; BmChr 23 fuses PxLG 23 and 30; and BmChr 24 fuses PxLG 24 and 31 (Table 3).

**Table 3:** Matching *Plutella xylostella* linkage groups with *Bombyx mori* chromosomes.

*B. mori*	*P. xylostella*	RAD hits to *B. mori* Chr.	Other matches	Maternal-derived	Paternal-derived	Uniref90 Hits
Chromosome	Linkage Group assignment	(TBLASTX<1E-10)	Chromsome(Hits)	RAD alleles	RAD alleles	(BLASTX<1E-10)
1	1	8	16(2)	104	147	15
2	2	6	17(1)	49	48	1
3	3	16	-	114	119	9
4	4	40	-	160	156	19
5	5	41	18(1),23(1)	190	152	29
6	6	13	-	89	101	14
7	7	1	23(1)	75	102	3
8	8	26	-	146	116	15
9	9	13	-	71	67	11
10	10	32	-	138	120	21
11	11	8	3(1),8(1),20(1)	69	63	9
12	12	18	-	98	90	10
13	13	17	-	112	105	14
14	14	7	-	60	80	10
15	15	33	-	129	158	25
16	16	40	-	150	118	19
17	17	23	-	142	88	15
18	18	3	-	76	115	10
19	19	8	16(1)	86	77	4
20	20	13	-	101	104	13
21	21	26	-	104	82	15
22	22	15	21(1)	98	61	6
23	23	18	-	108	94	10
24	24	6	22(1)	77	40	7
25	25	33	10(2)	133	132	18
26	26	4	-	75	49	3
27	27	17	-	105	71	10
28	28	6	13(1)	88	4	1
11	29	20	-	99	103	19
23	30	11	1(1)	78	85	6
24	**31**	5	7(1)	53	31	4
28 Chr.	31 Chr.	451 Hits		**3177**	**2878**	**365**

*P. xylostella* linkage groups reassigned to *B. mori* chromosomes based on number of TBLASTX hits of RAD contigs. 19 linkage groups had hits to a single chromosome. The remaining 12 linkage groups were assigned to the chromosome with the majority of hits. Other hits are shown in the table above. Maternal-derived candidate RAD allele counts are sums of the counts for the two segregation patterns for each linkage group shown in Table 2. Uniref90 hits are hits to unique proteins for RAD contigs.

### Genotyping Control Offspring and Identifying the Spinosad Resistance Chromosome

A chromosome carrying the spinosad resistance gene mutation was previously identified using AFLP markers [15]. Although only 20 backcross progeny were ultimately used in this study, a perfect correlation between PxLG5 and the expected spinosad resistance chromosome was detected. Of 190 RAD alleles assigned to PxLG5, 82 were present in all spinosad-treated surviving offspring and six untreated controls, indicating linkage to the chromosome encoding resistance. The remaining 108 RAD alleles were absent from all spinosad-treated individuals and present in the remaining three controls, indicating that this maternally derived chromosome encodes wild-type susceptibility to spinosad, and that these RAD alleles could be used to determine the previously unknown genotypes of the control offspring. This chromosome contained the highest number of mapped RAD clusters (n = 190), providing an extensive sequence dataset within this chromosome.

### Identification of sex chromosomes and sex-linked markers

Backcross progeny were sexed prior to DNA isolation (see Table 1). The sexes of the progeny matched the segregation patterns for one *P. xylostella* chromosome, which is syntenic with *B. mori* chromosome 1 (the *B. mori* Z chromosome [20]). Of 104 maternal-derived RAD alleles on PxLG1, 39 were present in female offspring alone, and were therefore W-linked, whereas 65 were present in male offspring alone, and therefore Z-linked (see Table 2). 147 paternal-derived RAD alleles were associated with PxLG1 Z-linked maternal-derived RAD alleles by sequence homology (see below). 15 protein-coding genes were associated with PxLG1 based on UniRef90 hits by the associated RAD contig (Table S1), and all of these genes were associated with Z-linked markers. No genes were associated with the W chromosome. Of the 15 genes, 8 have BLAST annotations to *B. mori* sex chromosome scaffolds, according to the Silkworm Genome Database (silkdb.org), two of which have previously been identified as sex-linked genes in Lepidoptera (Catalase [21], Stretchin-MLCK [22]).

### Linkage map of the *P. xylostella* genome

The experimental cross analysed here was intended to associate RAD alleles with chromosomes, and in particular, identify sequenced markers on the chromosome encoding spinosad resistance. Nonetheless, despite the small number of individuals sampled we were able to construct a linkage map of the *P. xylostella* genome with paternally derived RAD alleles. In total, 4,042 paternally derived candidate RAD alleles segregated among the progeny. Using allelic homology with the 3,177 maternally derived markers, the paternal markers were assigned to chromosomal linkage groups, where possible. Candidate RAD alleles were considered allelic between the parents provided that a) there were three or fewer base differences in the 40 base forward sequence and b) each paternal RAD allele matched either i) a single maternally derived RAD allele or ii) multiple RAD alleles on the same chromosome. Markers with multiple matches to multiple chromosomes (or no chromosome matches) were not assigned to a chromosome.

Once a subset of the paternal candidate RAD alleles had been assigned to chromosomes, the segregation patterns of all paternal markers were analyzed. Candidate RAD alleles that showed a segregation pattern identical to that of a previously chromosome-assigned marker were also assigned to that chromosome. Candidate RAD alleles displaying the inverse of these segregation patterns were also included. Finally paternally derived candidate RAD alleles with segregation patterns that differed by only one genotype from a chromosome-assigned marker were linked to that chromosome.

To minimize the likelihood of erroneous markers contributing to the linkage map, a robust criterion was established: each locus had to contain three or more distinct RAD alleles. Segregation patterns with less than three RAD alleles often had questionable support and so were discarded. Valid loci containing one or two RAD alleles could be manually identified, however a criterion of at least three markers per loci was sufficient for our purposes.

**Figure 2:**
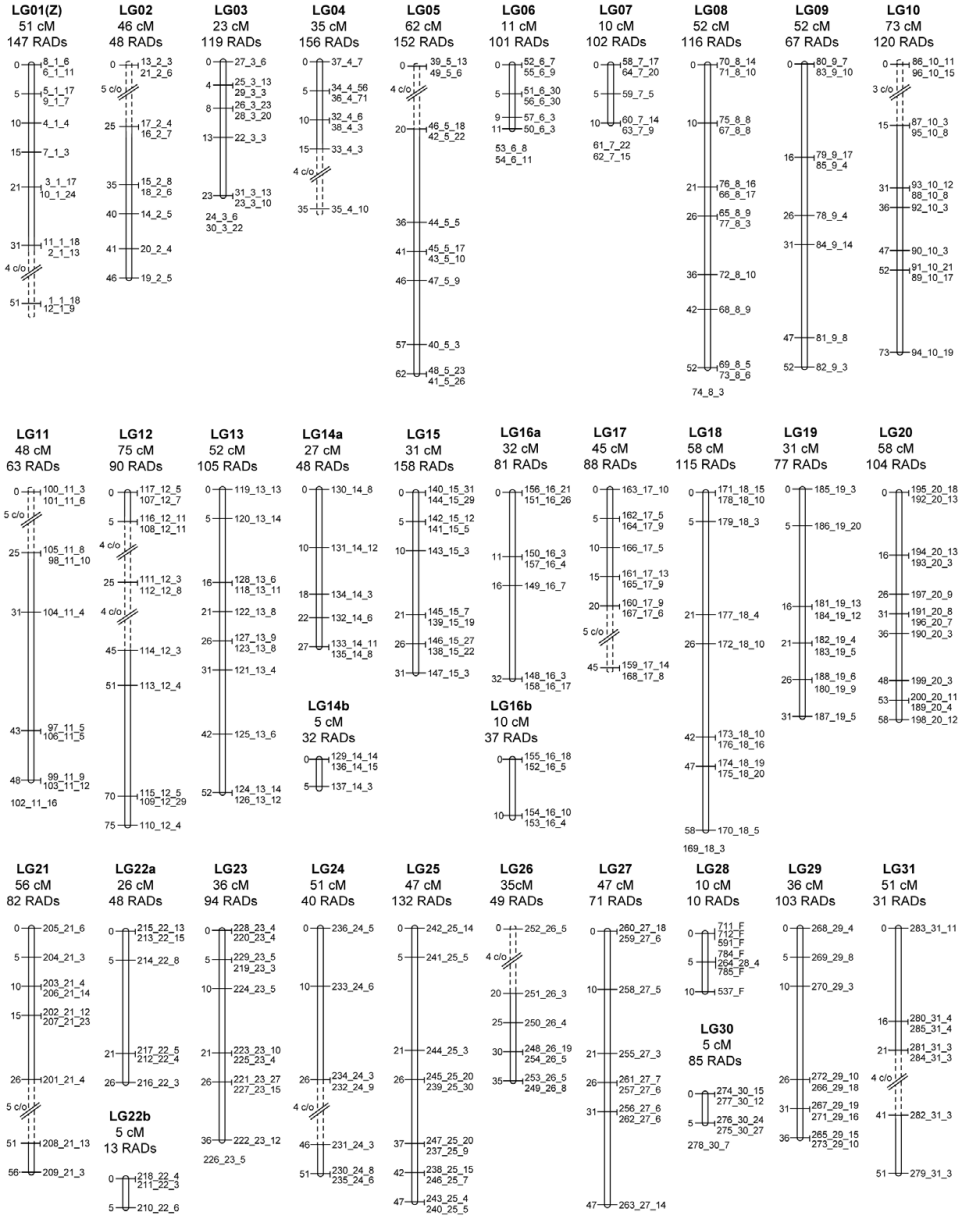
Linkage map of the *Plutella xylostella* (n = 31) genome. This was inferred from 2,878 RAD alleles collapsed into 285 discrete RAD markers. Each linkage group contains between 10 and 158 RAD alleles (labelled RADs) and the total map length is 1,292 cM. Each RAD marker is labelled with three numbers (i_ii_iii) corresponding to (i) the RAD marker (1–285), (ii) the chromosome number (1–31) and (iii) the number of RAD alleles at that marker. Linkage groups 1–28 are homologous to the *B. mori* (n = 28) chromosome numbering system, and LG29, LG30 and LG31 represent fusions to chromosomes 11, 23 and 24 respectively in *B. mori*. Dashed lines represent manual linkages inferred from 3, 4 or 5 genotype differences that were otherwise left ungrouped due to small sample size. As 20 progeny were used to construct the map, distances were approximated as 5 cM (1/20) per 1 crossing-over (c/o) event. On chromosomes 14, 16 and 22, markers formed two distinct groups and may be separated by regions of high recombination rates or chromosomal assignment error. In total, 11 of the 285 RAD markers could not be confidently assigned to their predicted chromosome. Linkage group 28 contained only four RAD markers at a single locus. Six additional markers were identified for this chromosome using JoinMap 3.0, from the remaining paternal markers not assigned to linkage groups.

Of the 4,042 paternally inherited and segregating candidate RAD alleles, 2,878 met this criterion. This set further collapsed into 285 distinct segregation patterns, or markers, containing between 3 and 71 RAD alleles at each position. Of these 2,878 RAD alleles, the associated RAD contigs of 718 hit the *B. mori* genome using TBLASTX, with 636 (89%) having at least one hit to their assigned chromosome. Linkage groups were assembled independently (JoinMap 3.0), producing a map length of 1292 cM (Figure 2, Table S2). Using measurements on nuclei stained with propidium iodide, we estimated the *P. xylostella* haploid genome size to be 339.4 (+/−1.1) Mb for homogametic males and 338.7 (+/−1.1) Mb for heterogametic females. This implies a physical map distance of 262.7 kb/cM in males.

### RAD contigs containing protein coding regions

RAD contigs were compared to the UniRef90 protein database using BLASTX to predict the number of chromosome linked RAD markers that are likely to be parts of protein-coding genes. Using an expect value significance cut-off of 1E-10, 282 (8.8%) of the 3,177 maternally derived RAD contigs and 231 (8.0%) of the 2,878 paternally derived RAD contigs are predicted to derive from protein-coding genes. As a proportion of markers were allelic between the cross parents, there were hits to 365 unique proteins (Table 4, Table S1).

**Table 4:** Oligonucleotide primers and adapters for RAD Sequencing.

Name	Sequence
P1-FOR-xxxxx	5′-Phos-AATGATACGGCGACCACCGAGATCTACACTCTTTCCCTACACGACGCTCTTCCGATCTxxxxxTGC*A-3′
P1-REV-xxxxx	5′-Phos-xxxxxAGATCGGAAGAGCGTCGTGTAGGGAAAGAGTGTAGATCTCGGTGGTCGCCGTATCAT*T-3′
P2-PE-FOR	5′-Phos-GATCGGAAGAGCGGTTCAGCAGGAATGCCGAGACCGATCAGAACAA-3′
P2-PE-REV	5′-Phos-CAAGCAGAAGACGGCATACGAGATCGGTCTCGGCATTCCTGCTGAACCGCTCTTCCGATC*T-3′
P1-PCR	5′-AATGATACGGCGACCACCGA-3′
P2-PCR	5′-CAAGCAGAAGACGGCATACGA-3′

xxxxx  =  MID Sequence (see Table 1).

*  =  phosphorothioate linkage.

## Discussion

Understanding the molecular control of novel phenotypes in complex organisms is a central goal of modern evolutionary biology. Identifying the general region of a genome containing an adaptive locus is often the first critical phase in this process that ultimately aims to pinpoint the causal sequence under selection. However, technical limitations have made this a considerable challenge in most species. Restriction-site associated DNA (RAD) sequencing presents a powerful approach to dramatically improve molecular marker density across genomes and considerably accelerate the identification of genomic regions linked to traits of interest. This technique has evolved from pioneering studies utilizing microarray hybridization techniques to score presence/absence of marker regions adjacent to restriction sites in the genome [23], to exploit next-generation techniques to directly sequence these regions [6]. RAD tags from a species can be mapped directly onto its reference genome sequence [9], however since none is available for *P. xylostella* we used paired-end sequences adjacent to the RAD tags for interspecific comparisons to the genome sequence of the model lepidopteran *Bombyx mori*. Here, using a single lane of Illumina GAIIx sequencing, we have identified over 8,000 candidate loci with about 14,000 alleles. The number of loci identified is larger than the 6,652 expected from a simple Poisson model of SbfI site frequency in the 339Mb *P. xylostella* genome, likely reflecting the inadequacy of this model in a complex, repeat-rich, eukaryote genome. These RAD-derived molecular markers have been assigned to chromosomal locations in the genome of the insect pest *P. xylostella*.

Due to a lack of crossing over in female Lepidoptera, we were able to confidently establish a set of 3,177 maternally inherited, chromosome-specific RAD markers identified by their segregation pattern among backcross progeny. Each of the 31 chromosomes contained 49–190 sequenced markers and, overall, 8% of the contigs associated with mapped RAD alleles showed homology to predicted proteins. From just a limited number of backcross progeny, we accurately mapped a Mendelian spinosad insecticide resistance trait to chromosome 5. The resistance chromosome produced the greatest number of maternally derived RAD markers (190), although it is unclear whether this is due to the chromosome size or physical linkage to the resistance locus. The spinosad resistant Pearl strain was previously crossed to susceptible strain Geneva88 to select for survival on an artificial diet and then reselected for spinosad resistance to produce the mapping strain, Pearl-Sel. The effect of this laboratory selection prior to the Pearl-Sel x Geneva88 backcross presented here meant that many polymorphisms were shared by cross parents, except those at or perhaps near to the resistance locus itself. This may have increased the number of markers that could be mapped on the resistance chromosome.

The maternally derived paired-end data allow a comprehensive genome wide comparison of synteny with the sequenced genome of *B. mori* [18]. This demonstrates a high degree of conservation of synteny between these species, such that chromosomal orthologues can be predicted with high confidence. As karyotypes of these two species differ by three chromosomes (n = 28 and n = 31), a perfect 1∶1 assignment was not expected. We identified homology between *P. xylostella* linkage groups and *B. mori* chromosomes, including the three putative fusion or breakage events. This high degree of synteny conservation at the level of whole chromosomes is consistent with previous analyses within the Macrolepidoptera, and suggests that the conservation seen previously between butterflies and *B. mori* [18], [24] is also true more widely in the Lepidoptera. Using assembled paired-end contigs for comparative genome analysis should have a considerable advantage over using short forward reads [11], particularly for non-model organisms.

Linkage maps have been produced using RAD sequence data in barley [11] and perennial ryegrass [25], which both have extensive genomic resources. Our experiment was not designed for construction of a linkage map, as too few individuals were sampled to robustly map markers to chromosomal positions. Nonetheless, the extensive sampling of the genome provided by the RAD markers enabled us to construct a linkage map based on recombinational distances between paternally inherited RAD markers. The paternal dataset of 2,878 RAD markers was limited to those with patterns of segregation observed three or more times and showing allelic homology to one of the 3,177 chromosome specific maternal markers. The genomic linkage map predicts a total map length of 1292 cM (Figure 2), similar to other lepidopteran linkage maps including *Heliconius melpomene* (1616 cM) [4], *B. mori* (between 1305 cM and 3229 cM) [20], [26], and *Bicyclus anynana* (between 1352 cM –1642 cM) [24], [27]. CentiMorgan distances separating markers can vary markedly, depending upon both mapping program and criteria. For example in *B. anynana,* Van't Hof (2008) calculated total linkage distances of 1354 cM and 1873 cM from the same data set using two different mapping programs.

The methods used for this study were suitable for our purposes but may not be appropriate for RAD studies in other species with different aims. We have provided information on the effect of using different numbers of mismatches and fragment count thresholds for our data (Tables S3 and S4) and recommend that others choose parameters suitable for their chosen experiment. The crude normalisation applied here (see Methods and Figure S1) allowed us to resolve chromosome prints cleanly but retained a large number of alleles resulting from sequencing error that were filtered using other criteria. Accurate normalisation of the curves shown in Figure S1 is clearly amenable to a machine learning approach, and we are continuing to address this problem.

The strength of RAD-based linkage maps is the volume of accountable genomic sequence. Traditional linkage maps based on anonymous markers are useful in determining rates of crossing over, or focusing on a specific locus. However, the anonymity of these markers limits the utility of such maps for further studies, and they provide no information on comparative genomics. More recently, a genetic linkage map for *Bicyclus anynana* was constructed based on EST sequences, but required costly prior identification of SNPs before mapping could be carried out [24]. In contrast, we here present a map for *Plutella xylostella* based on RAD alleles, large numbers of which can be readily identified as orthologues of known genes in reference genomes. Presence/absence patterns of RAD alleles furnish the basic data for linkage map construction, SNP variation within the 40-base RAD alleles enables integration of paternally- and maternally-informative linkage maps, and longer sequences adjacent to the RAD alleles provide enough information for cross-species comparisons. This will provide a wealth of genome sequence data for future gene-finding and genomic studies in this economically important pest species. Furthermore, our dataset demonstrates the feasibility of this approach for future genomic studies of any non-model organism.

## Materials and Methods

### Terminology

In what follows, a read is the raw sequence determined from the P1 adapter, containing the captured restriction site overhang, obtained on an Illumina sequencing instrument. The sequence read from the P2 adapter at the other end of the DNA fragment is called the paired-end read. A RAD locus is a region downstream of a restriction site. Each RAD locus contains one or more RAD alleles, depending on whether the locus is homozygous or heterozygous within and across individuals. RAD loci and RAD alleles are initially described as candidates to indicate that further error correction (such as removal of repeats and RAD alleles appearing in only one individual) will take place downstream during processing. A RAD contig is an assembly of the paired-ends for a single RAD allele into a long contiguous sequence. Each allele will have a read count (the number of reads associated with the allele) and a fragment count (the number of unique paired-end reads associated with the allele, assumed to represent the number of DNA fragments for this allele in the initial sheared genomic DNA sample). A segregation pattern is a pattern of presence/absence of a particular RAD allele across all individuals in the sample. A RAD marker is a segregation pattern of alleles at a locus used in the construction of the linkage map.

### Insects and Crosses

The spinosad-resistant strain Pearl was collected from Hawaii in 2001. A resistant derivative, Pearl-Sel, was isolated after crossing Pearl to a sensitive line (Geneva88) to facilitate laboratory adaptation. The reference spinosad-sensitive strain, Geneva88, was collected in 1988 and reared in the laboratory without exposure to insecticide. Pearl-Sel shows >1000 fold resistance to spinosad compared to Geneva88 [14]. Crosses were performed by mating a Pearl-Sel male with a Geneva88 female then backcrossing a single F_1_ female to a Pearl-Sel male. Approximately 60 backcross progeny were treated with a discriminating dose of spinosad (10 ppm) and, as resistance is recessive, survivors were inferred to be homozygous for the spinosad resistance allele. Some backcross progeny were reared without exposure to spinosad as controls and were either homozygous or heterozygous for the resistance allele [15].

### RAD Library Preparation

After sexing, single adult moths were homogenised in DNA isolation buffer and genomic DNA purified using three phenol extractions followed by a single chloroform extraction [28]. RNA was digested using RNase A, DNA precipitated and quantified using a NanoDrop. A paired-end RAD library was constructed using a protocol adapted from Baird *et al.* (2008). DNA samples from the backcross father, F_1_ mother (400 ng DNA each), 10 untreated controls and 12 bioassay survivors (200 ng each) were used to produce a single Illumina sequencing library.

Genomic DNA from each of the 24 library individuals was digested separately for 30 minutes at 37°C, in 50 µL reactions with 15 units of SbfI (NEB). The enzyme was heat inactivated at 65°C for 20 minutes. A different P1 adapter, each with a unique 5 bp molecular identifying sequence (MID) (Table 4), was then ligated to a designated individual (0.5 µL of 100 nM adapter, 1 µL 100 mM rATP, 1 µL NEB buffer 2, 0.5 µL T4 Ligase (NEB) with 400,000 cohesive end units/mL) by incubating for 2 hours at room temperature, then overnight at 4°C. The T4 ligase was heat deactivated at 65°C for 20 minutes. Samples were pooled (30 µL each) and a maximum of 300 µL aliquoted into 1.7 ml lo-bind tubes (Eppendorf). DNA was sheared using a Bioruptor set to high, for 8 minutes (30 s on/30 s off). Sheared DNA was purified with a Qiagen PCR cleanup column and eluted in 30 µL of buffer EB. The entire sample was size separated using gel electrophoresis (0.5X TBE, 1.2% agarose) and a DNA fraction corresponding to 300–700 bp was excised with a clean scalpel blade. The gel slice was purified with a Qiagen column and eluted in 20 µL of water.

Fragment ends were repaired using the Quick Blunting Kit (NEB) (19 µL DNA, 2.5 µL 10X buffer, 2.5 µL dNTP, 1 µL enzyme), incubated at room temperature for 30 minutes, purified with MinElute Reaction Cleanup Kit (Qiagen) and eluted in 16 µL water. dATP overhangs were added to the DNA using 15 µL of purified library template, dATP (1 µL 100 mM), 15 units Klenow exo- (NEB) and 2 µL NEB buffer 2. The reaction was incubated at 37°C for 30 minutes, then purified with a MinElute column and eluted in 21 µL of water.

Paired-end P2 adapter, containing T overhangs, was prepared by combining HPLC purified oligonucleotides P2-PE-FOR and P2-PE-REV (Table 4) in 1X annealing buffer, heating to 95°C and gradually allowing to cool. P2 adapter was ligated to 20 µL of sheared, size-selected, P1-ligated and pooled DNA template with 5 µL of 2 µM adapter, 1 µL of 100mM rATP, 3 µL NEB buffer 2 and 0.5 µL of 400,000 cohesive end unit/mL T4 DNA ligase in total reaction of 30 µL. The ligation was incubated overnight at 4°C then DNA purified with a MinElute column and eluted in 50 µL of buffer EB (Qiagen). PCR enrichment of the library was performed in seven 50 µL PCR reactions (2.5 µL template, 25 µL Phusion Flash High-Fidelity PCR Master Mix and 2.5 µL of P1-PCR [10 mM] and P2-PCR [10 mM] (Table 4) primer and 17.5 µL water). Cycling conditions were; 98°C for 30 s then 18 cycles of 98°C for 10 s, 65°C for 30 s, 72°C for 30 s, and a final extension at 72°C for 5 minutes. PCR amplicons 350–700 bases long were then size extracted using gel electrophoresis (0.5X TBE, 1.2% agarose). The RAD library was sequenced using an Illumina GAIIx instrument following standard protocols [10], and is available via the Sequence Read Archive through accession number ERP000449 (http://www.ebi.ac.uk/ena/data/view/ERP000449).

### Generating candidate RAD loci and RAD alleles

Raw Illumina reads from the P1 adapter were separated into pools according to the MID assigned to each individual sample in the RAD library. The 5 base MID and 6 base partial restriction site were removed from each 51 base read, leaving 40 bases of downstream sequence. For each individual, candidate RAD loci were identified and candidate RAD alleles called within loci as follows.

Identical reads were collapsed into unique sequences, with median quality values calculated for each unique sequence, and singleton reads discarded. Uniques less than a threshold distance apart were grouped together, with each group of uniques treated as a candidate RAD locus. Uniques were separated into different groups if they were more than a distance of 7 bases apart, allowing for sequence diversity and sequencing error between uniques within a group. This is likely to be an overestimate of sequence diversity in a real biological dataset, as it does not take repeat content into account, but a more liberal approach to clustering is preferred because there are many criteria by which clusters can be corrected at later stages. In fact, increasing the number of mismatches above three for this dataset has a minor effect on the number of candidate loci and alleles present in more than one individual in the final marker set (see Table S3). The distance between uniques was calculated by adding the differences between uniques and weighting each base difference by the probability that the two bases are called accurately (based on the median quality score for each base).

Each group of unique sequences was considered to be a candidate RAD locus. The set of uniques will include not only real RAD alleles but also sequencing errors. To correct these errors, for each possible base at each position in the locus, the mean quality score was calculated across all uniques. Bases that appeared in only one unique were rejected as likely errors. Uniques were then clustered together into candidate RAD alleles, merging uniques that were identical except for bases whose average qualities fell below a chosen quality threshold (in this case, Q = 20).

Candidate RAD loci and RAD alleles were defined based on the read containing the restriction site overhang, ignoring the paired-end read. Once RAD alleles were defined, the paired-end reads were grouped together by RAD allele. For each RAD allele, duplicate paired-end reads were collapsed into unique sequences, with the number of unique sequences taken to represent the number of DNA fragments in the original sample. These fragment counts were used instead of read counts in the following analysis.

Candidate RAD loci were then merged across all individuals if they shared a RAD allele in common, with 3 mismatches allowed between RAD alleles (Table S4). Fragment counts were crudely normalized for each individual, so thresholds could be applied over all samples. Scale factors were derived for each individual, using the mother *m* as a standard (scale factor 1), based on the curves shown in Figure S1. Ignoring the peak and long tail of each curve, the minimum and maximum of the curves were estimated manually for each individual *i*. The scale factor for individual *i* was calculated as *SF_i_* = (*min_m_*/*min_i_* + *max_m_*/*max_i_*)/2. The number of fragments for each candidate allele for individual *i* was then multiplied by *SF_i_* before thresholds were applied. RAD alleles with less than 3 fragments or only appearing in a single individual were discarded (see Table S4 and Discussion for more on thresholds and normalisation methods).

For each RAD allele, paired-end reads for that RAD allele were pooled together across all individuals and assembled using VelvetOptimiser version 2.1.4 [29] and velvet version 1.0.02 (http://bioinformatics.net.au/software.velvetoptimiser.shtml), using the ‘max’ optimisation function, which aims to generate the longest contig possible. The RAD contig produced does not overlap the associated RAD allele since the smallest fragment formed by shearing (approx 300 bp) is longer than the length of the read plus the paired-end read.

A general version of this pipeline has been implemented in the open source toolkit RADtools (http://www.radseq.info).

### Sequence comparison

The assembled RAD contigs were compared using the BLAST suite of programs (NCBI BLAST+ Version 2.2.23 [30] to the Uniref90 protein database [31], downloaded on 13 October 2010, and the assembled genome of *Bombyx mori*, (Integrated sequences uploaded 30 September 2008, downloaded 10 February 2009, from the Silkworm Genome Research Program, sgp.dna.affrc.go.jp [32] to identify potentially coding RAD contigs, and homologues in the sequenced lepidopteran genome.

### Linkage map construction

RAD loci heterozygous in the maternal parent were assigned to linkage groups corresponding to chromosomes, based on complete linkage. As crossing-over between non-sister chromatids only occurs during spermatogenesis in Lepidoptera, segregating RAD alleles from the father were used for linkage mapping. Of 4,042 paternally derived candidate RAD alleles, 702 could be assigned to a chromosome based on alleleism with the maternally derived dataset. The presence/absence segregating patterns for RAD alleles with allelic matches were then used as chromosome-anchored paternal RAD alleles to recover other candidate RAD alleles with identical patterns. In total, 2,878 paternal RAD alleles were recovered and these clustered into 285 distinct segregation patterns, hereafter called RAD markers, with each RAD marker containing a minimum of 3 RAD alleles, and this set of RAD markers was used for linkage map construction. Chromosomes were assembled separately by first grouping RAD markers with LOD>2, and then inferring chromosomal linkage maps with a LOD>1 and theta>0.4 using JoinMap 3.0 (Kyazma). Due to the small number of progeny analyzed (20 individuals) some chromosomes contained multiple linkage groups, or RAD markers that did not group together. In these cases, raw genotypes were manually analyzed to assess likely order. A maximum of 5/20 genotype differences (representing 5 crossing over events) were considered, with distance approximation of 1 event ∼ 5cM. Markers with more than 5 recombinants were not grouped together and are presented as distinct linkage groups for a particular chromosome.

### Genome size estimations

The genome size of *P. xylostella* was estimated by comparing red fluorescence from propidium iodide-stained nuclei isolated from brain tissue of single adults to that produced by co-prepared nuclei from the head of a single *Drosophila melanogaster* adult (1C = 175 Mb) using flow cytometry [4].

### Accession Numbers

The dataset for this study has been submitted to the Sequence Read Archive under accession number ERP000449.

## Supporting Information

Figure S1 **Normalisation of fragment counts.** Numbers of unique paired-end fragments per candidate allele are plotted against number of candidate alleles, for the mother, father and one backcross individual, control 07. For example, the mother has 149 candidate alleles with 6 fragments each and 259 candidate alleles with 27 fragments each. Both the axes are truncated, obscuring a large peak of alleles with three fragments or less (likely to be sequencing errors) and a long tail of alleles with eighty fragments or more (expected to be repeat clusters). Each curve shows a trough (marked with a circle) and a peak (marked with an arrowhead) which represent min_i_ and max_i_ respectively for each individual i (see Methods).
(PDF)

Table S1 **Uniref90 hits to** ***P. xylostella*** **chromosomes.** RAD allele sequences are listed with the best Uniref90 hit to their RAD contig. E-values are for TBLASTX. Alleles are listed as Mother (M) or Father (F) derived.
(XLS)

Table S2 **Dataset for linkage map construction.** 2,878 paternally derived RAD alleles were collapsed into 285 discrete RAD markers and used for linkage map construction (Figure 2). The forward sequence read and normalized sequence counts are provided for each individual, along with their segregating pattern across 20 backcross progeny in JoinMap 3.0 format (bold text). Each RAD marker (bold) and RAD allele is labelled with a set of three numbers corresponding to (i) the RAD marker (1-285), (ii) the chromosome number (1-31) and (iii) the number of alleles contributing to the marker (bold text) or the paternally derived allele number, between (1-2878).
(XLS)

Table S3 **Effect of grouping uniques into loci with different numbers of mismatches.** Reads for each individual were processed with the RADtools pipeline, allowing different numbers of mismatches between uniques when clustering them into candidate loci. The resulting candidate loci for each individual were then clustered together using the RADmarkers tool, with no thresholding of read or tag count and no mismatches allowed between sequences, only clustering together loci with identical alleles across individuals. After clustering, the loci present in only one individual were removed, as they are likely to be sequencing error. The numbers of loci and alleles fall sharply when using three mismatches instead of one mismatch, but only show small decreases as more than three mismatches are used.
(PDF)

Table S4 **Effects of normalisation, varying fragment count threshold and number of mismatches allowed during clustering with RADtools.** Loci with normalised fragment counts (A) and raw fragment counts (B) were clustered, allowing 0-6 mismatches and accepting only alleles with fragment counts equal to or above thresholds 1-6 (ie only alleles with at least the threshold number of fragments were allowed). All loci appearing in one individual only were discarded.
(PDF)
