# Supplementary figures and images for: Linkage Mapping and Comparative Genomics Using Next-Generation RAD Sequencing of a Non-Model Organism
Source: PLoS One. 2011 Apr 26;6(4):e19315. doi: 10.1371/journal.pone.0019315 (PMC3082572; doi:10.1371/journal.pone.0019315)

Figure S1

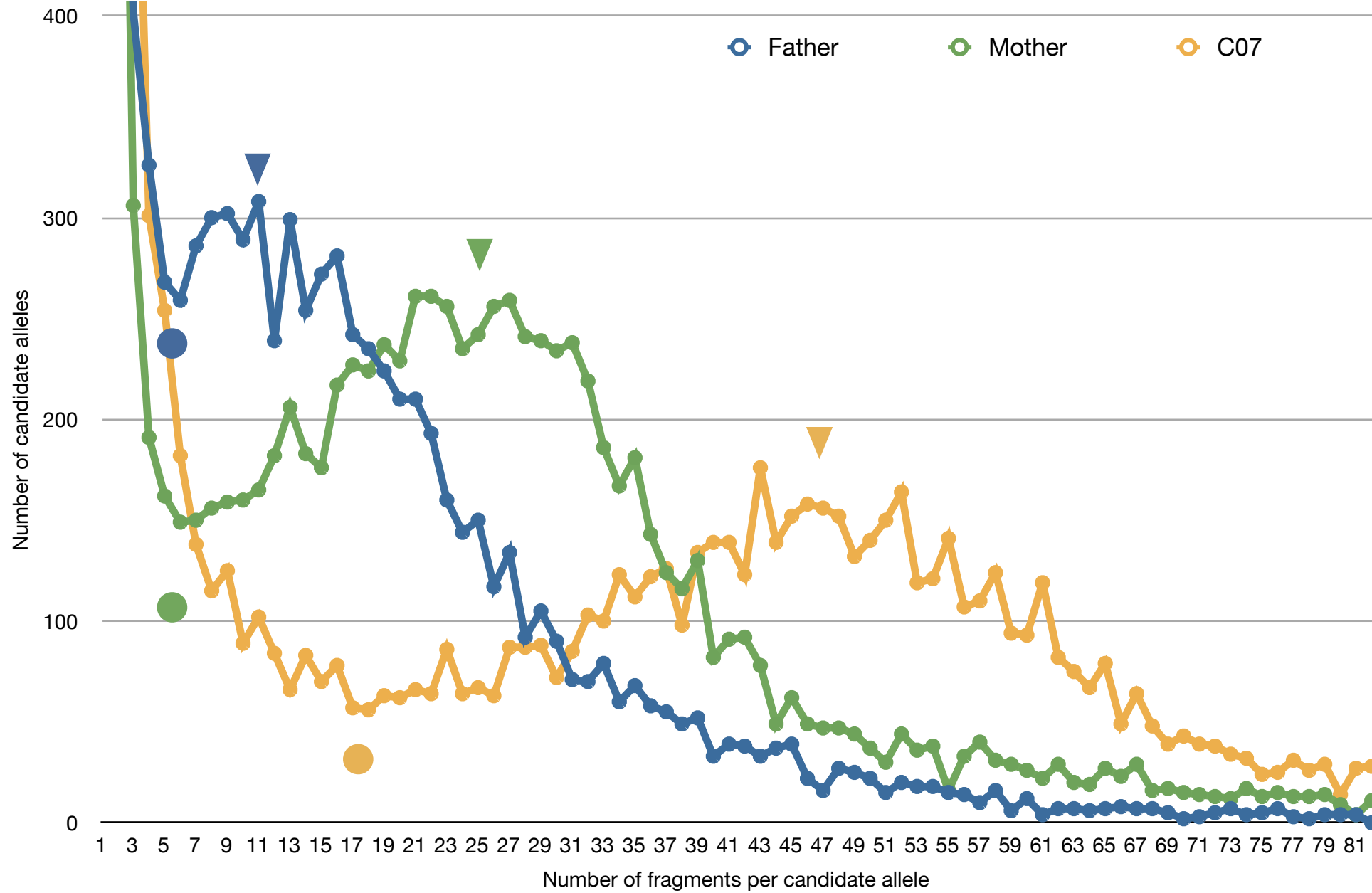

Supplement: Figure S1 — Normalisation of fragment counts. Numbers of unique paired-end fragments per candidate allele are plotted against number of candidate alleles, for the mother, father and one backcross individual, control 07. For example, the mother has 149 candidate alleles with 6 fragments each and 259 candidate alleles with 27 fragments each. Both the axes are truncated, obscuring a large peak of alleles with three fragments or less (likely to be sequencing errors) and a long tail of alleles with eighty fragments or more (expected to be repeat clusters). Each curve shows a trough (marked with a circle) and a peak (marked with an arrowhead) which represent mini and maxi respectively for each individual i (see Methods). (PDF) [file pone.0019315.s001.pdf]
