# Supplementary material for: Linkage Mapping and Comparative Genomics Using Next-Generation RAD Sequencing of a Non-Model Organism
Source: PLoS One. 2011 Apr 26;6(4):e19315. doi: 10.1371/journal.pone.0019315 (PMC3082572; doi:10.1371/journal.pone.0019315)
Supplement: Table S3 — Effect of grouping uniques into loci with different numbers of mismatches. Reads for each individual were processed with the RADtools pipeline, allowing different numbers of mismatches between uniques when clustering them into candidate loci. The resulting candidate loci for each individual were then clustered together using the RADmarkers tool, with no thresholding of read or tag count and no mismatches allowed between sequences, only clustering together loci with identical alleles across individuals. After clustering, the loci present in only one individual were removed, as they are likely to be sequencing error. The numbers of loci and alleles fall sharply when using three mismatches instead of one mismatch, but only show small decreases as more than three mismatches are used. (PDF) [file pone.0019315.s004.pdf]

Table S3

Mismatches	1	2	3	4	5	6	7	8	9	10
**All loci**	61,395	41,288	33,240	29,212	27,049	25,894	24,523	23,758	23,073	22,588
**All alleles**	66,805	50,801	45,191	42,554	41,194	40,549	39,050	38,403	37,748	37,435
**Loci present in more than one individual**	16,583	13,390	12,212	11,642	11,359	11,183	11,025	10,870	10,760	10,643
**% of loci in more than one individual clustered with one mismatch**	100	81	74	70	68	67	66	66	65	64
**Alleles present in more than one individual**	19,274	17,609	17,004	16,789	16,704	16,658	16,585	16,517	16,478	16,439
**% of alleles in more than one individual clustered with one mismatch**	100	91	88	87	87	86	86	86	85	85
