# Supplementary material for: Linkage Mapping and Comparative Genomics Using Next-Generation RAD Sequencing of a Non-Model Organism
Source: PLoS One. 2011 Apr 26;6(4):e19315. doi: 10.1371/journal.pone.0019315 (PMC3082572; doi:10.1371/journal.pone.0019315)
Supplement: Table S4 — Effects of normalisation, varying fragment count threshold and number of mismatches allowed during clustering with RADtools. Loci with normalised fragment counts (A) and raw fragment counts (B) were clustered, allowing 0-6 mismatches and accepting only alleles with fragment counts equal to or above thresholds 1-6 (ie only alleles with at least the threshold number of fragments were allowed). All loci appearing in one individual only were discarded. (PDF) [file pone.0019315.s005.pdf]

Table S4

A. Loci with normalised fragment counts

Fragment Count Threshold	Number of Mismatches						
	0	1	2	3	4	5	6
>=1	10,385	9,252	8,971	8,813	8,700	8,600	8,532
>=2	9,826	8,933	8,662	8,517	8,407	8,312	8,241
>=3	9,502	8,756	8,483	8,342	8,234	8,140	8,066
>=4	9,232	8,555	8,295	8,149	8,045	7,959	7,891
>=5	8,975	8,332	8,084	7,947	7,847	7,763	7,696
>=6	8,770	8,156	7,917	7,785	7,692	7,615	7,548

B. Loci with raw fragment counts

Fragment Count Threshold	Number of Mismatches						
	0	1	2	3	4	5	6
>=1	11,025	9,825	9,533	9,367	9,251	9,150	9,079
>=2	10,286	9,170	8,892	8,734	8,621	8,523	8,454
>=3	9,742	8,897	8,626	8,475	8,365	8,272	8,199
>=4	9,444	8,717	8,449	8,302	8,194	8,105	8,032
>=5	9,189	8,511	8,253	8,107	8,002	7,920	7,847
>=6	8,995	8,345	8,100	7,960	7,860	7,776	7,709
